# Multiscale microenvironmental perturbation of pluripotent stem cell fate and self-organization

**DOI:** 10.1038/srep44711

**Published:** 2017-03-17

**Authors:** Yoji Tabata, Matthias P. Lutolf

**Affiliations:** 1 Laboratory of Stem Cell Bioengineering, Institute of Bioengineering, School of Life Sciences and School of Engineering, École Polytechnique Fédérale de Lausanne (EPFL), CH-1015 Lausanne, Switzerland; 2 Institute of Chemical Sciences and Engineering, School of Basic Science, EPFL, CH-1015 Lausanne, Switzerland.

## Abstract

The combination of microfluidics with engineered three-dimensional (3D) matrices can bring new insights into the fate regulation of stem cells and their self-organization into organoids. Although there has been progress in 3D stem cell culturing, most existing *in vitro* methodologies do not allow for mimicking of the spatiotemporal heterogeneity of stimuli that drive morphogenetic processes *in vivo*. To address this, we present a perfusion-free microchip concept for the *in vitro* 3D perturbation of stem cell fate. Stem cells are encapsulated in a hydrogel compartment that is flanked by open reservoirs for the diffusion-driven generation of biomolecule gradients. Juxtaposing additional compartments bearing supportive cells enables investigating the influence of long range cell-cell communication. We explore the utility of the microchips in manipulating early fate choices and self-organizing characteristics of 3D-cultured mouse embryonic stem cells (mESCs) under neural differentiation conditions and exposure to gradients of leukemia inhibitory factor (LIF). mESCs respond to LIF gradients in a spatially dependent manner. At higher LIF concentrations, multicellular colonies maintain pluripotency in contrast, at lower concentrations, mESCs develop into apicobasally polarized epithelial cysts. This versatile system can help to systematically explore the role of multifactorial microenvironments in promoting self-patterning of various stem cell types.

Since the discovery of stem cells, great progress has been made in understanding the molecular and cellular mechanisms that regulate the self-renewal and differentiation of these fascinating cells. In adult tissues, as well as in developing embryos, stem cell behavior is strongly influenced by extrinsic factors from the microenvironmental niche^1^,^2^. Because of the complexity of total organisms, it is challenging to elucidate the role of microenvironmental factors in regulating the fate of live stem cells directly *in vivo*. Therefore, the development of *in vitro* models that can simulate key characteristics of native stem cell niches has become a promising alternative. Such models must take into account both the biophysical and biochemical properties of the extracellular matrix (ECM), the presence of soluble bioactive molecules, and the presence of other cell types that play a role in supporting stem cells through either direct cell–cell communication or long-range, diffusible signals^3^.

Numerous biomaterials have been designed as cell culture substrates, offering properties that are more physiological than conventional plastic dishes. Besides having similar structural and mechanical properties compared to natural ECMs, synthetic hydrogels offer an unprecedented modularity and enable the fabrication of chemically defined microenvironments in a reproducible and customizable manner^4^,^5^. Indeed, synthetic hydrogels have been engineered to support the three-dimensional (3D) culture of various stem cell types; in some cases, stem cells have even been coaxed into self-patterning multicellular constructs that resemble primitive tissues^6^. However, in contrast to conventional, static cultures in hydrogels, *in vivo* processes involving stem cells are triggered by a highly spatially and temporally complex display of various microenvironmental signals^1^,^2^,^7^,^8^,^9^. Therefore, to study more complex (patho-)physiological processes at the tissue or organ level, there is a crucial need for cell culture platforms that permit better control of biological signals in space *and* time.

Soft lithography–based microfluidic chips offer exciting possibilities for building advanced cell culture systems^10^. For example, through controlled delivery of nanoliter-scale fluids, cells in a defined location on a chip can be exposed to a desired signal at a specific time (e.g. refs 11, 12, 13). However, existing microfluidic systems are often poorly suited for the long-term maintenance of stem cells and their development into organoids, as the cellular substrates in these devices lack instructive signals and there is limited space for tissue development. Furthermore, cell behavior may be compromised in microfluidic culture because of the presence of shear stresses^14^, the depletion of important autocrine factors^15^ and medium evaporation^16^. Finally, existing microfluidic culture systems often require dedicated equipment and skills, which hampers their widespread use in biological laboratories.

To address these shortcomings, we present an easy-to-use microchip concept that enables cells cultured within desired hydrogels to be exposed to spatiotemporally modular and well-controlled biomolecule distributions. Optionally, by using chemically defined hydrogels and appropriate bioconjugation strategies, biomolecules can be tethered to hydrogel networks and presented in a graded manner. Additionally, integration of a hydrogel compartment containing a supporting cell type (e.g. ‘feeder’ cells for the maintenance of stem cells), enables studying the influence of long range cell-cell communication in a spatially dependent manner. Since the operation of the microchip does not rely on active perfusion, cells are not exposed to fluid flow, resulting in much higher cell viability due to an accumulation of important autocrine and paracrine factors in the cell culture chamber. We employed this platform for the 3D culture of mouse embryonic stem cells (mESCs) under neural induction conditions, when their differentiation was locally perturbed by exposure to gradients of soluble, cell secreted and gel-immobilized leukemia inhibitory factor (LIF), a self-renewal factor. We demonstrated that 3D-cultured single mESCs under neural induction conditions strongly respond to the local LIF concentration: The maintenance or loss of pluripotency and the establishment of apicobasally polarized colonies were found to be dependent on the relative position of the mESC-derived colonies in relation to the LIF gradient. We think that this tool is well suited for *in vitro* study of the role of extrinsic factors driving early morphogenetic processes in stem cell–mediated organogenesis.

## Results

### Design, fabrication, and characterization of a biomicrofluidic chip

**Figure 1:**
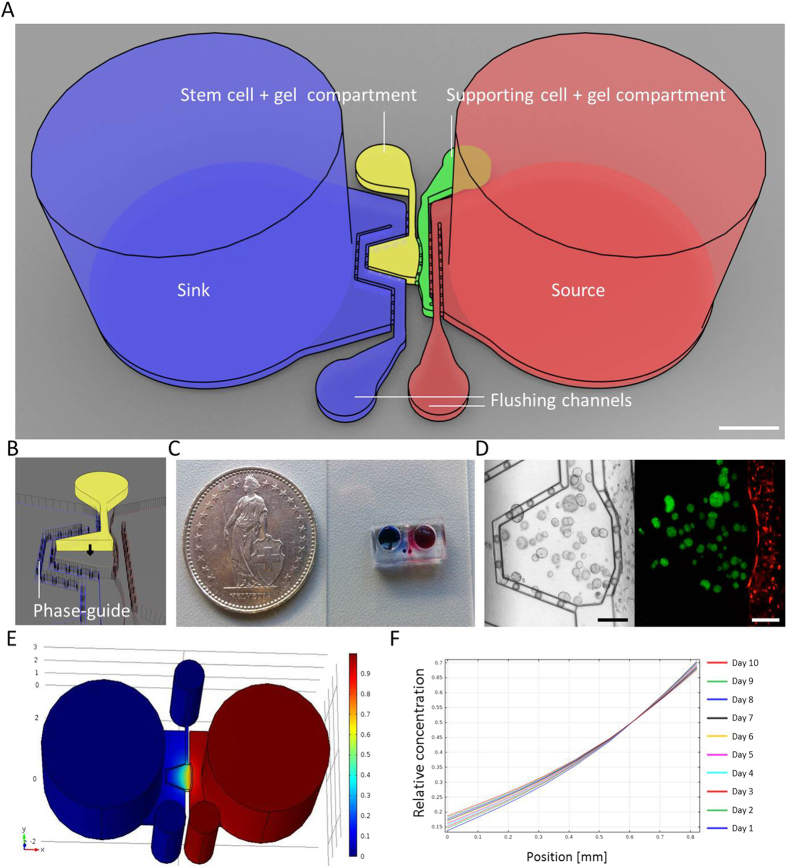
Design and characterization of the microfabricated platform. (**A**) Schematic representation of the microfabricated device. The device is composed of single or multiple cell-laden hydrogel compartments flanked by two open reservoirs. The scheme shows the device with two chambers. Scale bar indicates 1 mm. (**B**) Compartments are delimited by phase-guiding features that enable independent loading without spillage. (**C**) The two reservoirs can be used as source (red) and sink (blue) of a molecule of interest. The molecule will diffuse from the source to the sink while forming gradual distribution within the hydrogel chamber. (**D**) Multiple compartments allow for patterned co-culture of multiple cell types. Rex1::GFP mouse embryonic stem cells (green) and tomato reporter fibroblasts (red) were co-cultured for 3 days. Scale bars indicate 200 μm. (**E**) Computational simulation of molecule distribution in the microfabricated device with a single chamber. Simulation was performed with dedicated software (COMSOL Multiphysics, COMSOL AB). The device model was reasonably simplified, and molecular diffusivity in hydrogel was assumed to be equivalent to 0.4 relative to diffusivity in aqueous solution. (**F**) Simulative quantification of molecule distribution across the hydrogel compartment from day 1 to day 10. The estimated fold changes between the two extremities of the chamber were 5.1 and 3.6 at day 1 and day 10, respectively.

Our device was composed of one or two juxtaposed hydrogel compartments sandwiched between two open reservoirs (Fig. 1A,C). Integration of phase-guiding features^17^, a combination of continuous projection and pillars of regular intervals, maximized the hydrodynamic resistance at compartment boundaries and enabled casting cell-laden hydrogels in the dedicated compartments without any spillage (Fig. 1B). With the double-compartment platform, two different cell types could be juxtaposed into interconnected individual chambers (Fig. 1D), which could be independently loaded with the desired hydrogel formulations. The side reservoirs were interconnected through the hydrogel compartment(s) and prevent convective interactions, resulting in mass transfer mediated by Fickian diffusion. We postulated that diffusion-controlled gradients of soluble molecules could be established by simply filling the source reservoir with a medium formulation of interest. Indeed, computational simulations confirmed the establishment and stability of quasi-linear gradients over 10 days, while the relative variation of the profile did not exceed 20% (Fig. 1E,F). For this molecular model, the fold change in concentration between the two extremities of the chamber ranged from 6 to 4 at day 1 and 10, respectively. Because of the small dimensions of the hydrogel compartment, mass transfer flux of the molecules across the compartment should not significantly affect the functional integrity of the source/sink pair during the timespan required for an experiment. Notably, the diffusion of biomolecules should also be controllable by the crosslinking density of the hydrogels, which tends to hinder the diffusion of higher molecular weight molecules, such as large proteins^18^. Consequently, the larger difference between the diffusivity of the molecule in liquid and in the permeable hydrogel network resulted in a slower but steeper dynamic gradient, as demonstrated by the simulations.

**Figure 2:**
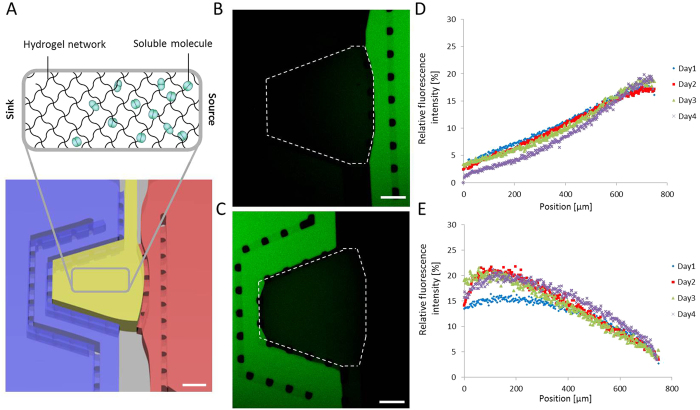
Generation of stable gradients of soluble molecules via source–sink diffusion. (**A**) Schematic representation of the diffusion of soluble molecules through the hydrogel compartment. Molecules diffused from the source reservoir (red) to the sink reservoir (blue) while forming a gradient of concentration. Fluorescently labeled LIF solution was loaded onto either side of the platform in the right (**B**) or left (**C**) reservoir and incubated for 4 days without renewal. Evolution of fluorescence intensity across the hydrogel compartment was monitored daily via confocal microscopy. Quantification of the fluorescence intensity displays gradual distribution of the molecule across the hydrogel compartment. (**D**) and (**E**) are the quantification of (**B**) and (**C**), respectively. Intensity was normalized relative to the intensity in the source and sink reservoirs and displayed as the mean value of three replicates. Scale bars indicate 200 μm.

We experimentally tested the predictions from the computational simulations. Fluorescently labeled LIF was used as a model protein to visualize the profile of the gradient and monitor its stability over up to 4 days (Fig. 2A). These experiments revealed that the shape and kinetics of gradient formation are influenced by the dimensions of the hydrogel–reservoir interfaces. The dimensions of the two interfaces are unequal and thus application of molecules through the smaller interface made the system reach equilibrium faster, generating a quasi-linear gradient profile (Fig. 2B,D). In contrast, application through the larger interface retarded the speed of stabilization, generating an arc-type profile (Fig. 2C,E). In the case of the smaller interface, the fold change in concentration between the two extremities of the chamber was found to be 6.8 ± 2.2, in line with the simulative analysis. In the case of the larger interface, the baseline concentration was found to be slightly higher, resulting in a lower fold change (4.8 ± 0.9 fold) between the two sides. For both conformations, the maximal fluorescence intensity measured in the hydrogel compartments was equivalent to approximately 20% of the source signal. Notably, due to the small dimensions of the hydrogel compartment and the absence of tubing, only a few tens of microliters of biomolecule solution was required to maintain stable gradients over several days. This represents a considerable reduction in reagent consumption compared to conventional fluidic-based systems, which typically require hundreds of microliters up to a few milliliters^12^,^19^.

### Establishment of matrix-tethered biomolecule gradients

**Figure 3:**
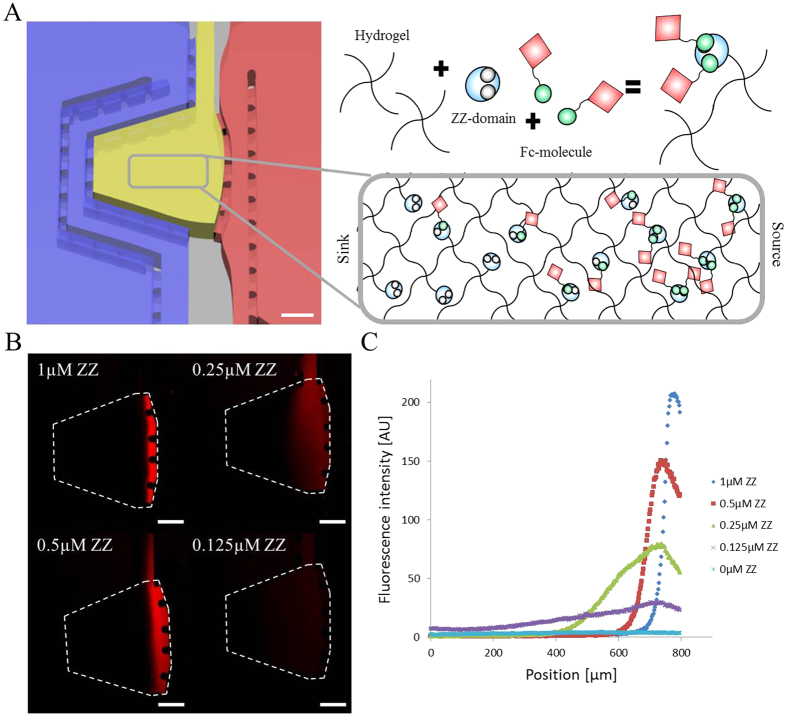
Generation of gradients of immobilized biomolecules via Fc–ZZ coupling and source–sink diffusion. (**A**) Schematic representation of the gradual functionalization of hydrogel compartment. Fc-tagged molecules diffused and became trapped in the hydrogel compartment that was pre-functionalized with the ZZ domain. (**B**) The gradient profile is determined by the concentration of incorporated ZZ domain. Sharper profiles are obtained with a high relative concentration of the ZZ domain. Gradients were visualized at day 1 with confocal microscopy. Scale bars indicate 200 μm. (**C**) Observations were confirmed with quantification. Values are displayed as the mean value over three replicates.

In native tissues, many key regulatory proteins are attached to the crosslinked ECM through electrostatic and other intermolecular interactions. This is important not only for their spatial localization but also their bioactivity^20^. To recapitulate this aspect of ECM biology in our microchip platform, we utilized a selective and generic protein immobilization scheme based on the interaction of protein A, an immunoglobulin-binding bacterial protein, with the Fc region of immunoglobulins, which has already been successfully explored for gradient generation^21^. To immobilize Fc-tagged proteins to 3D poly(ethylene glycol) (PEG)-based hydrogels, ZZ, a linker peptide containing two repeats of the synthetic protein A analog Z, was enzymatically crosslinked to the PEG networks, providing high-affinity binding sites for Fc-tagged bioactive ligands^22^. Indeed, a highly localized functionalization of our hydrogel could be obtained by exposing the central chamber to a source of Fc-tagged LIF (Fc-LIF; Fig. 3A) which was visualized by fluorescently labeling the molecule. On lateral diffusion into the hydrogel compartment, Fc-LIF was captured by the ZZ domains, resulting in the establishment of a LIF gradient. The local concentration of trapped molecules was strongly dependent on the concentration of available ZZ domains in the PEG network, as well as the time of Fc-LIF exposure (Fig. 3B,C). A low ZZ-domain concentration resulted in a relatively flat and linear gradient, whereas a high ZZ-domain concentration resulted in the generation of a highly localized distribution in very close proximity to the source. The high affinity of ZZ–Fc interaction ensured that the tethered gradients would be stable for at least 4 days (Fig. S2).

### Microfluidic manipulation of mESC fate and self-organization via graded display of LIF

Recent work by several groups highlighted the remarkable propensity of stem cells to self-organize *in vitro* in 3D culture, recapitulating key aspects of mammalian organogenesis^23^. For example, it has been shown that single mESCs embedded in Matrigel^TM^ or synthetic PEG-based gels are able to expand and organize themselves into apicobasally polarized cysts^6^,^24^. As a proof of principle, we investigated the possibility of growing single mESCs in 3D culture into multicellular colonies, locally stimulating them to acquire specific identities and potentially self-organize in a disparate manner. We hypothesized that mESCs grown in neural induction medium and exposed to gradients of the self-renewal factor LIF could be promoted to either give rise to colonies of pure stem cells or else exit pluripotency to form early epithelial tissue-like structures, depending on LIF concentration, that is, their spatial localization.

**Figure 4:**
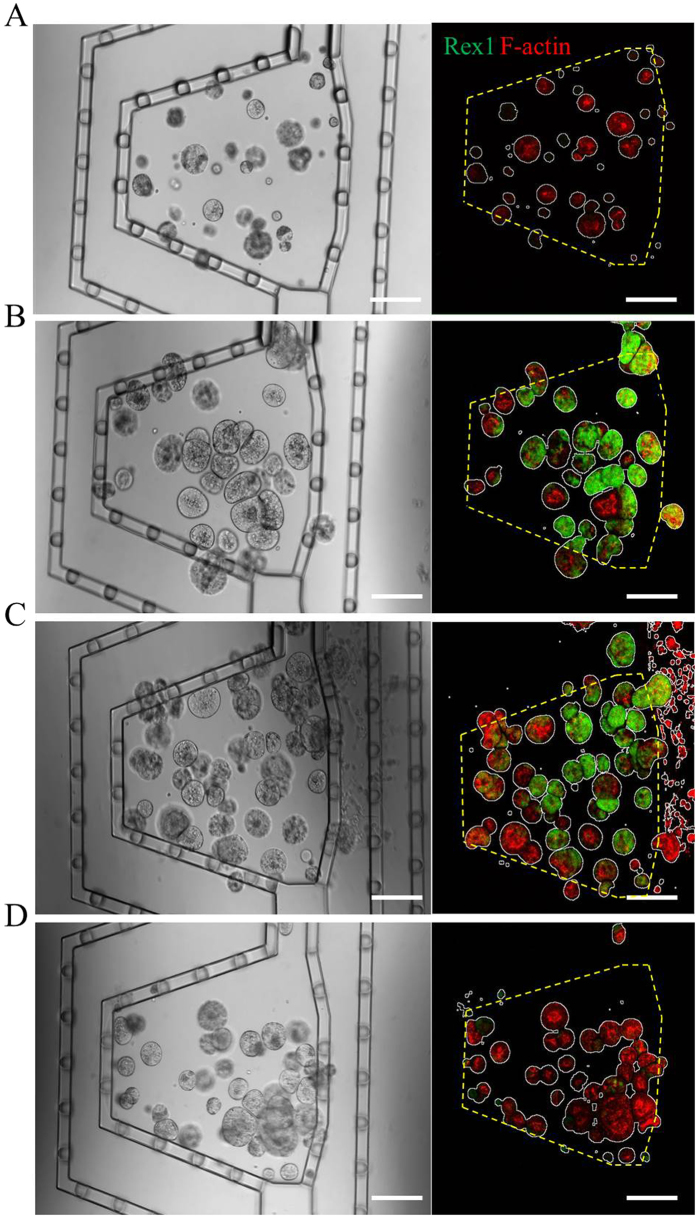
Exposure of mESCs to various LIF gradients. mESCs were cultured for 4 days in the device and their fate locally perturbed with three different formats of LIF-based stimulation. Cells were cultured (**A**) without or in presence of (**B**) the gradient of soluble LIF with source concentration of 20 ng/mL, (**C**) patterned SNLP cells that overexpress LIF with density of 1200 cells/μL, and (**D**) the gradient of immobilized LIF where the gel was treated with Fc-LIF for 8 hours. Gradual distribution of Rex1 (green) and F-actin polarization (red) can be observed in some conditions. White outlines indicate colonies stained with DAPI and yellow lines indicate borders of the hydrogel compartment. Scale bars indicate 200 μm.

To test this, Rex1::GFP mESCs that report the pluripotency state were encapsulated as single cells in laminin-containing PEG hydrogels casted in the microchip. Cells were cultured for 4 days in response to gradients of soluble LIF (Fig. 4), which were provided through diffusion from the source reservoir, secreted by co-cultured murine embryonic fibroblast feeder cells (SNLP 76/7-4)^25^, or gel tethered. In all experiments, mESCs showed good cell viability at day 2 (74.7 ± 3.4%), which was found to be significantly (*p* = 0.006) higher than the standard bulk 3D PEG hydrogel culture conditions (62.1 ± 2.3%). Single mESCs developed into large, clonally derived colonies in all conditions. After culturing, cells were fixed and stained with DAPI and phalloidin to quantify the extent of self-renewal (GFP expression), differentiation (loss of GFP), and apicobasal polarization (visualized by phalloidin-stained actin filaments).

**Figure 5:**
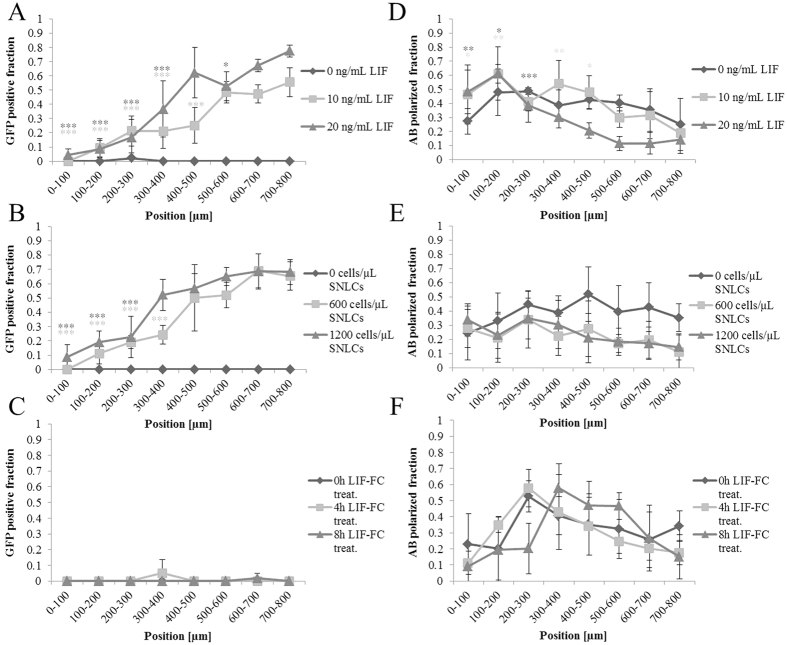
Quantification of mESC fate in response to various LIF gradients. Quantification of the GFP positive fraction of mESC colonies with respect to their position in the hydrogel compartment for the (**A**) soluble LIF gradient, (**B**) co-culture with patterned SNLP cells, and (**C**) immobilized LIF gradient condition. Quantification of the fraction of mESC colonies acquiring apicobasal (actin polarization) polarity for the (**D**) soluble LIF gradient, (**E**) secreted LIF gradient, and (**F**) tethered LIF gradient condition. Positions <0 μm and >800 μm correspond to the sink and source reservoirs, respectively. Quantification of each sector was compared with the value of the 700–800-μm sector with one-tailed analysis of variance. Means and standard deviations were derived from four independent experiments with three replicates each. *p < 0.05, **p < 0.01, and ***p < 0.001.

In all three experimental groups, by day 4 the “no stimulation” condition—corresponding to the cultures not comprising any LIF—resulted in smaller colony areas and complete down-regulation of green fluorescent protein (GFP), marking Rex1 expression, independent of the position of colonies within the cell culture chamber. The graded delivery of LIF led to mESC colony development of disparate and spatially dependent identity: Soluble (Figs 4A and 5A) and cell-secreted LIF (Figs 4B and 5B) gradients triggered colonies with a graded distribution of GFP expression, with the highest GFP intensity in the region closest to the LIF source. In contrast to the soluble LIF condition, the exact concentration of LIF in the co-culture condition is unknown as the optimal density of feeder cells has been empirically found. The fraction of GFP-positive colonies was found to be slightly higher throughout the culture chamber when the amount of LIF, or density of feeder cells, was doubled. Furthermore, actin polarization and loss of Rex1 correlated with a total or partial loss of GFP-positive cells within the colony, often in association with the emergence of actin polarization. Contrary to GFP expression, a higher fraction of polarized colonies was observed in the region furthest from the LIF source (Figs 4A and 5D). Notably, the growth and survival rates of the cells were also slightly higher near the LIF source (Fig. S3), likely because LIF not only represses commitment but also contributes to the survival and growth of mESCs^26^. Interestingly, in the co-culture condition, clonally derived mESC colonies exhibited similar apicobasal polarization frequency (Figs 4B and 5E) and higher growth rate independent of the position, likely because feeder cells release extra paracrine factors that may alter their morphologic transition. What is more, when cells were exposed to gel-immobilized LIF, we found that GFP expression was lost by day 4 (Figs 4C and 5C), and no localized actin polarization was observed (Figs 4C and 5F). Of note, increasing the concentration of gel-immobilized LIF up to 100 times compared to the soluble LIF condition did not improve the results (data not shown).

## Discussion

The long-term culture of PSCs in state-of-the-art microfluidic chips is a significant challenge due to reduced auto/paracrine signaling^15^; shear stress^14^; and instability of medium conditions, including pH, osmolarity and oxygen content^16^. Our platform offers microchip culture conditions that are as close as possible to more favorable bulk cultures, while enabling precise cell manipulation through (*i*) localized exposure to soluble molecules, (*ii*) localized functionalization of the matrix, and (*iii*) integration of supportive cell populations.

The usage of reservoirs allows for the generation of stable biomolecule gradients, as shown by both simulative and experimental studies. In fact, minimization of the mass-transfer area between the two reservoirs is key to ensuring the long-term stability of the system. Simulative data over 10 days shows that the concentration of the source and sink lost and gained 8% of the initial source concentration (data not shown). Such small variation could be achieved even without any reservoir renewal, implying that the mass-transfer flux is small enough to keep the reservoirs functional for a long time. The long term integrity of the reservoirs could be further improved by increasing the volumes (e.g. making the chip thicker), or by regularly renewing its content. However, in our setup, the maximal concentration difference from source to sink was about 4–5 fold for molecules of a size of about 60 kDa. In that regard, microdevices integrating flow can offer a wider concentration range (e.g. ref. 27), although only a few of these systems have been applied for stem cell culture^28^,^29^, and they are usually restricted to two-dimensional (2D) settings with the presence of serum or a high cell density needed to allow for acceptable cell viability. Instead, in our system, cell viability does not differ from static 3D culturing methods despite an extremely low cell number and the reduction of serum (not shown).

Other groups have reported on the integration of hydrogels into microfluidic devices to generate tissue-like structures with specific spatial arrangement of cells and bioactive factors^30^. However, to maintain the long-term localization of bioactive components in these systems, it was necessary to integrate fluid paths in a closed system. Other, more complex systems have been developed to study the dose-dependent responses of stem cells^31^,^32^, but in these systems, each condition needs to be evaluated separately, making it impossible to assess the effects of gradual concentration changes on stem cell fate and patterning.

Our experiments with mESCs showed that the platform ensures satisfactory stem cell growth and that spatially heterogeneous responses could be induced in sub-millimeter environments by controlling the local concentration of a signaling molecule. Cells in proximity to the LIF source maintained Rex1 expression, marking the ground state of mESCs^33^, whereas those close to the sink lost it. The loss of Rex1 in ESC colonies seemed to be accompanied by an acquisition of apicobasal polarity; this was in line with our previous observations on the clonal formation of neuro-epithelial cysts obtained in N2B27 in different 3D matrices^6^,^24^. Based on previous 2D experiments, LIF alone is not sufficient to maintain the ground state of mESCs^26^,^33^. However, we and others have found that a 3D milieu may promote pluripotency through spatial confinement and enhanced cell–cell interactions^34^. Indeed, encapsulated mESCs can retain Oct4 for prolonged periods of time even after the removal of LIF^35^.

Our experiments showed that the ECM-tethered LIF is not effective despite the quantitative equivalence with other conditions. In this situation, a finite amount of LIF may be locally immobilized with no replenishment, diverging from the two previous conditions, in which LIF was continuously supplied. This observation raises several hypotheses, as follows:

1. The tight interaction of ZZ/Fc hinders the efficient formation of LIF/LIFR complex;
2. Consumption of LIF leads to its depletion over time; and
3. Tethered LIF is exclusively available to the cells on the surface of the colonies, which would preclude the colonies from being uniformly exposed to LIF during the entire culture period, resulting in the loss of Rex1.

Nevertheless, the larger size of colonies in the high-tethered LIF region (Fig. S3) suggests that there is a temporal activity of LIF that contributes to spatial difference for colony size but cannot maintain pluripotency throughout the culture period.

A potential limitation of the current set-up is the inability of controlling the cell localization within the culture chamber. Cells are randomly seeded which causes heterogeneous local density, potentially influencing ESC fate^36^. This problem can be overcome by measuring sufficiently large numbers of independent samples.

Early examples of the application of microfluidic gradient makers to the localized control and patterning of PSCs were performed in the context of 2D culture conditions^12^,^21^. Recently, more physiologically relevant 3D hydrogel-based gradient markers have been reported. For example, we had developed an agarose-based micro-well substrate equipped with a perfusion system to instruct embryoid bodies^29^. Moreover, Kamm and colleagues introduced a microfluidic platform for locally directing the neural fate of PSCs embedded in a collagen matrix^37^. Here, defects related to the usage of fluids were counterbalanced by the relatively large dimension associated with high cell density, as well as the usage of a xeno-derived matrix and the high concentration of serum replacement. The application of such devices may be restricted to a few specific systems. Moreover, these platforms may necessitate a complex manufacturing process, and the category of stimulation appears restricted to soluble molecules.

The local tethering of bioactive molecules in our system allows a wide range of concentrations to be attained across the hydrogel chamber. This approach is especially appropriate for bioactive components that must be immobilized to be effective, such as molecules involved in regulating cell adhesion and mechanotransduction^38^. Devices for immobilizing gradients of bioactive molecules have recently been reported for both 2D^21^ and 3D^39^ systems. Such devices, however, require laboratories equipped for microfabrication, whereas our device allows gradients of tethered molecules to be created in a considerably easier manner.

Taking the results together, we have presented a novel microengineered platform for the 3D culture of stem cells within engineered microenvironments in which (stem) cell activity can be locally controlled by three categories of signals, namely soluble molecules, cell-secreted molecules and ECM-immobilized molecules. Each signal can be manipulated and integrated into the system independently. Our characterization of biomolecular diffusion demonstrated that the molecular distribution can be tightly controlled in long-term experiments. In contrast to existing gradient generators, our approach does not rely on active perfusion, making it highly cell and user friendly. We demonstrated here that the gradual distribution of biomolecules can be exploited to induce biological effects that are dependent on the local concentration acting on cells. This platform should be useful for systematically probing and manipulating the fate and self-patterning of other stem cell types.

## Methods

### Microdevice design and mask fabrication

Design of the microfabricated device is shown in (Fig. 1A). The device was composed of the main 200 μm-high and 800 μm-high cell-laden hydrogel compartment flanked by two 4 mm-wide open medium reservoirs. Each compartment was interconnected while being partitioned by the 50 μm-thick phase-guiding features that enable compartments to be loaded separately with dedicated materials without any spillage. Additional 100 μm-wide channels were placed between the hydrogel compartments and reservoirs to remove residual air after loading and polymerization of the hydrogel. The two reservoirs could be used as a pair of source/sink for generating the gradual distribution of bioactive molecules. The device layout was drawn with dedicated software (CleWin, Phoenix Software) and printed on a resist-and chrome-coated glass mask (Nanofilm) via high-resolution laser-based method (VPG200, Heidelberg instruments). Unexposed photoresist was then removed with a developer (DV10, Süss MicroTec) and the underlying chrome layer etched with an acid/oxidizer solution of perchloric acid, cerium ammonium nitrate and water. Finally, the resulting mask was developed with TechniStrip P1316 (Microchemicals) to remove the residual resist and extensively washed with ultra-pure water.

### Soft lithography and PDMS molding

The microfabricated platform was fabricated using conventional lithography methods and poly(dimethysiloxane) (PDMS) replica molding. The mold was made from multiple-layered, epoxy-based negative photoresist using the design previously described. 150 μm thick layer of SU8 GM1075 (Gersteltec) photoresist was spin-coated and subsequently baked at 130 °C onto a pre-dehydrated silicon wafer using a negative resist coater (LMS200, Sawatec). The wafer was then aligned and exposed to UV (MA6/BA6 Süss MicroTec) through the first mask. After baking it at 95 °C, a 50μm-thick second layer of SU8 CM1070 (Gersteltec) was spin-coated and exposed to UV through the second mask that was carefully aligned to the wafer using dedicated alignment marks. After the post-exposure bake at 95 °C for 2 hours, the wafer was developed with propylene glycol monomethyl ether acetate (Sigma) and baked again at 135 °C overnight. The thickness of the total SU8 layer was confirmed with a surface profilometer (Dektak XT, Bruker). The wafer was then silanized with trichloro (1 H, 1 H, 2 H, 2 H-perfluorooctyl) silane (Sigma) overnight. The microstructured wafer was finally used for PDMS (Sylgard 184, Dow Corning) molding. The resulting PDMS replica was cut and punched with biopsy punchers of appropriate size (Kai medical). Resulting chips were exposed to oxygen plasma (PDC Harrick) and irreversibly bonded onto a glass substrate. Chips were sterilized with UV and kept at 37 °C in humidified environment prior to their use.

### Computational simulation

The commercial modeling software COMSOL Multiphysics (COMSOL AB) was used to simulate the diffusion of soluble molecules within the microchip device that was reasonably simplified. The molecular diffusion coefficient in water of the soluble molecule with molecular weight of 70 kDa (D_w_ = 3.2 × 10^−7^ cm^2^/s) was estimated based on the reported diffusion coefficient of 70 kDa dextran^40^. The molecular diffusion coefficient in the permeable PEG hydrogel (D_g_ = 1.8 × 10^−7^ cm^2^/s) was experimentally derived as described elsewhere^41^. Simulative quantification of molecule distribution across the hydrogel compartment was performed for each day from day 1 to day 10.

### Cell culture

Rex1::GFP reporter mESC line (generously provided by Prof. Austin Smith, University of Cambridge) was used as the model for our fate decision assay. Rex1 is a marker of pluripotency exclusively expressed in naïve-state mESCs and thus is an adequate reporter for monitoring the *in vitro* maintenance of pluripotency. Cells were maintained on a gelatin-coated dish in N2B27 medium composed of DMEM-F12 supplemented with 20 mM HEPES, 0.1 mM non-essential amino acids, 1 mM sodium pyruvate, 0.1 mM beta-mercaptoethanol, N2 supplement, B27 supplement, and 100 units/mL penicillin + 100 μg/mL streptomycin (all purchased from Gibco) 3 μM CHIR99021 (Stemgent), 1 μM PD0325901 (Selleckchem), Fc-LIF (produced in the Protein Expression Core Facility of EPFL, estimated molecular weight: 60 kDa). Growth factors and small molecules were freshly added to the medium prior to use. Cells were maintained at 37 °C in 5% CO_2_ humidified air and passaged every 3 days by enzymatic treatment with Accutase (Gibco). SNLP 76/7-4 (ATCC) is a mouse embryonic fibroblast cell line engineered for LIF over-expression, widely used as feeders for maintaining pluripotent stem cells^25^. SNLP cells were cultured in DMEM supplemented with 10% FBS (both from Gibco). Cells were maintained at 37 °C in 5% CO_2_ humidified air and passaged every 3 days by enzymatic treatment with TrypLE (Gibco).

### PEG hydrogel preparation

PEG hydrogels were prepared as described elsewhere^42^. Briefly, 8-arm PEG vinylsulfone (20 kDa PEG-VS, NOF) was conjugated with FXIII-substrate peptides via Michael type reaction in 0.3 M triethanolamine (pH 8.0) at 37 °C for 2 h. Glutamine-containing peptide (NQ-EQVSPL-ERCG-NH2) and two types of lysine-containing peptide sequence, either MMP-sensitive (AcFK-GG-GPQGIWGQ-ERCG-NH2) or MMP-non-sensitive (AcFK-GG-GPQGIAGF-ERCG-NH2) (custom made by GLBiochem) were used in this reaction. After dialysis (Snake Skin, MWCO 10 kDa, Pierce) and lyophilization, NQ-PEG and different AcFK-MMP-PEG powders were re-suspended in a molar-equivalent ratio in tris-buffered saline solution (TBS, 50 mM Tris, 10 mM CaCl_2_, pH7.6) to form precursor solutions of degradable W-PEG and non-degradable AF-PEG hydrogels. Gelation occurs within few minutes upon the addition of 20 units/mL of FXIIIa, pre-activated with thrombin. To activate FXIIIa, 200 units/mL of fibrogammin P1250 (CSL Behring) were mixed with 20 units/mL of thrombin (Sigma-Aldrich) in presence of 2.5 mM CaCl_2_ at 37 °C for 30 minutes.

### Hydrogel loading and gradient characterization

Before loading the hydrogel, the open reservoirs were prefilled with TBS to humidify the device. 2% (w/v) AF-PEG solution was mixed with 10 Units/mL of FXIIIa and carefully loaded into the hydrogel compartment. Upon complete gelation, air was removed from the flushing channel to expose the hydrogel to the medium contained in the reservoirs. To generate a soluble gradient, either side of the compartment was exposed to a solution of 1 μM fluorescently labeled Fc-LIF. Fc-LIF was functionalized with Alexa488-NHS or Alexa546-NHS (Invitrogen), following the manufacturer’s instructions. To generate a tethered gradient, hydrogel precursor solutions were mixed with various concentrations (from 0 to 1 μM) of ZZ-domain (generously provided by Dr. Martin Ehrbar, University of Zurich). ZZ-domain is composed of transglutaminase substrate and two subsequent repeats of ProteinA. In this way, ZZ-domains are integrated into the PEG backbone, and its affinity for Fc-tagged molecules allows proteins to be tethered to the PEG hydrogel backbone^22^. The hydrogel compartment was exposed from one side to 1 μM fluorescently labeled Fc-LIF for 8 hours and then extensively washed with TBS such as to create an immobilized gradient of LIF. The device was kept at 37 °C under humidified air, and fluorescence was imaged daily by confocal microscopy (Zeiss LSM700 invert). 5 images were captured across a z-stack height of 200 μm that were then collapsed into a single image.

### On-chip local manipulation of mESCs

mESCs at a density of 500 cells/μL were loaded into the device with 2% AF-PEG hydrogel. The PEG hydrogel was supplemented with 100 μg/mL laminin (Invitrogen) and optionally with 1 nM of ZZ-domain for protein immobilization conditions. For co-culture condition, SNLP cells were cultured in the side compartment at a density of 0, 600 or 1200 cells/μL in 1.5% W-PEG supplemented with 100 μM RGD. Reservoir was filled with the N2B27 medium supplemented with 1% Knockout Serum Replacement (KSR) (Gibco). For the soluble LIF gradient condition, cells were exposed to 0, 10 or 20 ng/mL Fc-LIF from the source reservoir. For the tethered LIF gradient, hydrogel was locally treated from the source reservoir with 120 ng/mL Fc- for 0, 4 or 8 hours and then extensively washed with culture medium. Cells were cultured for 4 days and media reservoirs were renewed at day 2. At the end of the culture, cells were fixed with 4% PFA (Gibco) and stained with DAPI (Sigma-Aldrich) and Alexa635-phalloidin (Invitrogen).

### Imaging and image analysis

Imaging was carried out using a conventional confocal microscopy (Zeiss LSM700). Stained samples were imaged in the DAPI, Alexa 635 and GFP channels separately. A 10x objective was used to capture the entire chamber in a single field of view. 12 images were captured across a z-stack height of 200 μm that were then collapsed into a single image. Manual counting of the proportion of Rex1-positive, and AP-polarized colonies versus DAPI positive entities was performed.

### Cell viability assay

Cells were 3D cultured either on-chip or in hydrogel discs at a density of 500 cells/μL for 2 days in the maintenance medium. Calcein AM and ethidium homodimer-1 staining (Life Technologies) was uitilized following the manufactures’ instructions. Manual counting of the proportion of live versus dead cells was performed on three independent samples.

### Statistical analysis

Data were analzyed by using paired Student’s t-test using Graphpad Prism 6.0 statistical software (Graphpad Software). Statistical significance level were represented as *p < 0.05, **p < 0.01, and ***p < 0.001. Means and standard deviations were derived from four independent experiments with three replicates each.

## Additional Information

**How to cite this article:** Tabata, Y. and Lutolf, M. P. Multiscale microenvironmental perturbation of pluripotent stem cell fate and self-organization. *Sci. Rep.* **7**, 44711; doi: 10.1038/srep44711 (2017).

**Publisher's note:** Springer Nature remains neutral with regard to jurisdictional claims in published maps and institutional affiliations.

## References

[b1] Scadden D. T. The stem-cell niche as an entity of action. Nature 441, 1075–1079, doi: 10.1038/nature04957 (2006). 16810242

[b2] Wang L. D. & Wagers A. J. Dynamic niches in the origination and differentiation of haematopoietic stem cells. Nat Rev Mol Cell Biol 12, 643–655, doi: 10.1038/nrm3184 (2011). 21886187 PMC4040463

[b3] Lutolf M. P., Gilbert P. M. & Blau H. M. Designing materials to direct stem-cell fate. Nature 462, 433–441, doi: 10.1038/nature08602 (2009). 19940913 PMC2908011

[b4] Lutolf M. P. & Hubbell J. A. Synthetic biomaterials as instructive extracellular microenvironments for morphogenesis in tissue engineering. Nat Biotechnol 23, 47–55, doi: 10.1038/nbt1055 (2005). 15637621

[b5] Kyburz K. A. & Anseth K. S. Synthetic Mimics of the Extracellular Matrix: How Simple is Complex Enough? Annals of Biomedical Engineering 43, 489–500, doi: 10.1007/s10439-015-1297-4 (2015). 25753017 PMC4380864

[b6] Ranga A. . Neural tube morphogenesis in synthetic 3D microenvironments. Proc Natl Acad Sci USA, doi: 10.1073/pnas.1603529113 (2016). PMC5098636 27742791

[b7] Zeller R., Lopez-Rios J. & Zuniga A. Vertebrate limb bud development: moving towards integrative analysis of organogenesis. Nat Rev Genet 10, 845–858, doi: 10.1038/nrg2681 (2009). 19920852

[b8] Kicheva A. . Kinetics of morphogen gradient formation. Science 315, 521–525, doi: 10.1126/science.1135774 (2007). 17255514

[b9] Kosinski C. . Gene expression patterns of human colon tops and basal crypts and BMP antagonists as intestinal stem cell niche factors. Proc Natl Acad Sci USA 104, 15418–15423, doi: 10.1073/pnas.0707210104 (2007). 17881565 PMC2000506

[b10] El-Ali J., Sorger P. K. & Jensen K. F. Cells on chips. Nature 442, 403–411, doi: 10.1038/nature05063 (2006). 16871208

[b11] Lucchetta E. M., Lee J. H., Fu L. A., Patel N. H. & Ismagilov R. F. Dynamics of Drosophila embryonic patterning network perturbed in space and time using microfluidics. Nature 434, 1134–1138, doi: 10.1038/nature03509 (2005). 15858575 PMC2656922

[b12] Kawada J., Kimura H., Akutsu H., Sakai Y. & Fujii T. Spatiotemporally controlled delivery of soluble factors for stem cell differentiation. Lab on a chip 12, 4508–4515, doi: 10.1039/c2lc40268h (2012). 22968416

[b13] Torisawa Y. S. . Microfluidic platform for chemotaxis in gradients formed by CXCL12 source-sink cells. Integr Biol (Camb) 2, 680–686, doi: 10.1039/c0ib00041h (2010). 20871938 PMC4128891

[b14] Kshitiz Kim D. H., Beebe D. J. & Levchenko A. Micro- and nanoengineering for stem cell biology: the promise with a caution. Trends Biotechnol 29, 399–408, doi: 10.1016/j.tibtech.2011.03.006 (2011). 21549437 PMC3726268

[b15] Blagovic K., Kim L. L. Y. & Voldman J. Microfluidic Perfusion for Regulating Diffusible Signaling in Stem Cells. Plos One 6, doi: ARTN e22892 10.1371/journal.pone.0022892 (2011). PMC3150375 21829665

[b16] Halldorsson S., Lucumi E., Gomez-Sjoberg R. & Fleming R. M. Advantages and challenges of microfluidic cell culture in polydimethylsiloxane devices. Biosens Bioelectron 63, 218–231, doi: 10.1016/j.bios.2014.07.029 (2015). 25105943

[b17] Vulto P. . Phaseguides: a paradigm shift in microfluidic priming and emptying. Lab on a chip 11, 1596–1602, doi: 10.1039/c0lc00643b (2011). 21394334

[b18] Weber L. M., Lopez C. G. & Anseth K. S. Effects of PEG hydrogel crosslinking density on protein diffusion and encapsulated islet survival and function. J Biomed Mater Res A 90, 720–729, doi: 10.1002/jbm.a.32134 (2009). 18570315 PMC2913724

[b19] Toh Y. C. . A microfluidic 3D hepatocyte chip for drug toxicity testing. Lab on a chip 9, 2026–2035, doi: 10.1039/b900912d (2009). 19568671

[b20] Bonnans C., Chou J. & Werb Z. Remodelling the extracellular matrix in development and disease. Nature Reviews Molecular Cell Biology 15, 786–801, doi: 10.1038/nrm3904 (2014). 25415508 PMC4316204

[b21] Cosson S., Allazetta S. & Lutolf M. P. Patterning of cell-instructive hydrogels by hydrodynamic flow focusing. Lab on a chip 13, 2099–2105, doi: 10.1039/c3lc50219h (2013). 23598796

[b22] Lienemann P. S. . A Versatile Approach to Engineering Biomolecule-Presenting Cellular Microenvironments. Advanced Healthcare Materials 2, 292–296, doi: 10.1002/adhm.201200280 (2013). 23184806

[b23] Huch M. & Koo B. K. Modeling mouse and human development using organoid cultures. Development 142, 3113–3125, doi: 10.1242/dev.118570 (2015). 26395140

[b24] Meinhardt A. . 3D reconstitution of the patterned neural tube from embryonic stem cells. Stem Cell Reports 3, 987–999, doi: 10.1016/j.stemcr.2014.09.020 (2014). 25454634 PMC4264068

[b25] Williams R. L. . Myeloid leukaemia inhibitory factor maintains the developmental potential of embryonic stem cells. Nature 336, 684–687, doi: 10.1038/336684a0 (1988). 3143916

[b26] Ying Q. L. . The ground state of embryonic stem cell self-renewal. Nature 453, 519–523, doi: 10.1038/nature06968 (2008). 18497825 PMC5328678

[b27] Kim S., Kim H. J. & Jeon N. L. Biological applications of microfluidic gradient devices. Integrative Biology 2, 584–603, doi: 10.1039/c0ib00055h (2010). 20957276

[b28] Kawada J., Kimura H., Akutsu H., Sakai Y. & Fujii T. Spatiotemporally controlled delivery of soluble factors for stem cell differentiation. Lab Chip 12, 4508–4515, doi: 10.1039/c2lc40268h (2012). 22968416

[b29] Cosson S. & Lutolf M. P. Hydrogel microfluidics for the patterning of pluripotent stem cells. Scientific Reports 4, doi: Artn 4462 10.1038/Srep04462 (2014). PMC3964519 24662945

[b30] Shin Y. . Microfluidic assay for simultaneous culture of multiple cell types on surfaces or within hydrogels. Nat Protoc 7, 1247–1259, doi: 10.1038/nprot.2012.051 (2012). 22678430 PMC4035049

[b31] Occhetta P. . Towards Modeling Limb Development: High-Throughput Microfluidic Platform for 3D Mesenchymal Stromal Cell Cultures. Tissue Engineering Part A 21, S267–S267 (2015).

[b32] Lecault V. . High-throughput analysis of single hematopoietic stem cell proliferation in microfluidic cell culture arrays. Nature Methods 8, 581–U593, doi: 10.1038/Nmeth.1614 (2011). 21602799

[b33] Kalkan T. & Smith A. Mapping the route from naive pluripotency to lineage specification. Philosophical Transactions of the Royal Society B-Biological Sciences 369, doi: 10.1098/rstb.2013.0540 (2014). PMC4216463 25349449

[b34] Lei Y. G. & Schaffer D. V. A fully defined and scalable 3D culture system for human pluripotent stem cell expansion and differentiation. Proceedings of the National Academy of Sciences of the United States of America 110, E5039–E5048, doi: 10.1073/pnas.1309408110 (2013). 24248365 PMC3876251

[b35] Caiazzo M. . Defined three-dimensional microenvironments boost induction of pluripotency. Nature Materials 15, 344–+, doi: 10.1038/NMAT4536 (2016). 26752655

[b36] Ranga A. . 3D niche microarrays for systems-level analyses of cell fate. Nature communications 5, 4324, doi: 10.1038/ncomms5324 (2014). PMC4104440 25027775

[b37] Uzel S. G. . Simultaneous or Sequential Orthogonal Gradient Formation in a 3D Cell Culture Microfluidic Platform. Small 12, 612–622, doi: 10.1002/smll.201501905 (2016). 26619365 PMC4752442

[b38] Murphy W. L., McDevitt T. C. & Engler A. J. Materials as stem cell regulators. Nat Mater 13, 547–557, doi: 10.1038/nmat3937 (2014). 24845994 PMC4163547

[b39] Lienemann P. S. . Locally controlling mesenchymal stem cell morphogenesis by 3D PDGF-BB gradients towards the establishment of an *in vitro* perivascular niche. Integrative Biology 7, 101–111, doi: 10.1039/c4ib00152d (2015). 25385042

[b40] Arrio-Dupont M., Cribier S., Foucault G., Devaux P. F. & d’Albis A. Diffusion of fluorescently labeled macromolecules in cultured muscle cells. Biophysical journal 70, 2327–2332, doi: 10.1016/S0006-3495(96)79798-9 (1996). 9172756 PMC1225207

[b41] Brandenberg N. & Lutolf M. P. *In Situ* Patterning of Microfluidic Networks in 3D Cell-Laden Hydrogels. Adv Mater 28, 7450–7456, doi: 10.1002/adma.201601099 (2016). 27334545

[b42] Ehrbar M. . Biomolecular hydrogels formed and degraded via site-specific enzymatic reactions. Biomacromolecules 8, 3000–3007, doi: 10.1021/bm070228f (2007). 17883273

